# Critical importance of pH and collector type on the flotation of sphalerite and galena from a low-grade lead–zinc ore

**DOI:** 10.1038/s41598-021-82759-3

**Published:** 2021-02-04

**Authors:** Abdolrahim Foroutan, Majid Abbas Zadeh Haji Abadi, Yaser Kianinia, Mahdi Ghadiri

**Affiliations:** 1 Department of Research and Development, Bama Mining Company, Isfahan, 81747-54754 Iran; 2 Institute of Research and Development, Duy Tan University, Da Nang, 550000 Viet Nam; 3 The Faculty of Environment and Chemical Engineering, Duy Tan University, Da Nang, 550000 Viet Nam

**Keywords:** Environmental chemistry, Environmental impact

## Abstract

Collector type and pulp pH play an important role in the lead–zinc ore flotation process. In the current study, the effect of pulp pH and the collector type parameters on the galena and sphalerite flotation from a complex lead–zinc–iron ore was investigated. The ethyl xanthate and Aero 3418 collectors were used for lead flotation and Aero 3477 and amyl xanthate for zinc flotation. It was found that maximum lead grade could be achieved by using Aero 3418 as collector at pH 8. Also, iron and zinc recoveries and grades were increased in the lead concentrate at lower pH which caused zinc recovery reduction in the zinc concentrate and decrease the lead grade concentrate. Furthermore, the results showed that the maximum zinc grade and recovery of 42.9% and 76.7% were achieved at pH 6 in the presence of Aero 3477 as collector. For both collectors at pH 5, Zinc recovery was increased around 2–3%; however, the iron recovery was also increased at this pH which reduced the zinc concentrate quality. Finally, pH 8 and pH 6 were selected as optimum pH values for lead and zinc flotation circuits, respectively.

## Introduction

The Gushfil Lead–Zinc mine is located in the Irankuh Mining District, 20 km South West of Esfahan city in Iran. The Irankuh Mining District (IMD) is a part of the Malayer-Esfahan Metallogenic Belt between Sanandaj and Sirjan cities^1^. It has been proposed that the IMD is an epigenetic Pb, Zn, and Ba Mississippi Valley-Type (MVT) according to isotopic investigations^2^. Later, Hoseini-Dinani and Aftabi^3^ proposed an MVT-type genetic model based on the soil and host rock geochemical halos of Zn, Pb, Ag, Ba, Hg and Sb. Carbonate host rocks and Early Cretaceous clastic detrital form the main part of The Gushfil deposit. The major sulphide mineralization occurs in the hanging-wall of the Gushfil-Baghabrisham fault. In sulphide ore facies, there are different types of textures including massive, vein-veinlet, semi-massive, colloform, framboidal, and laminated^4^.

**Figure 1:**
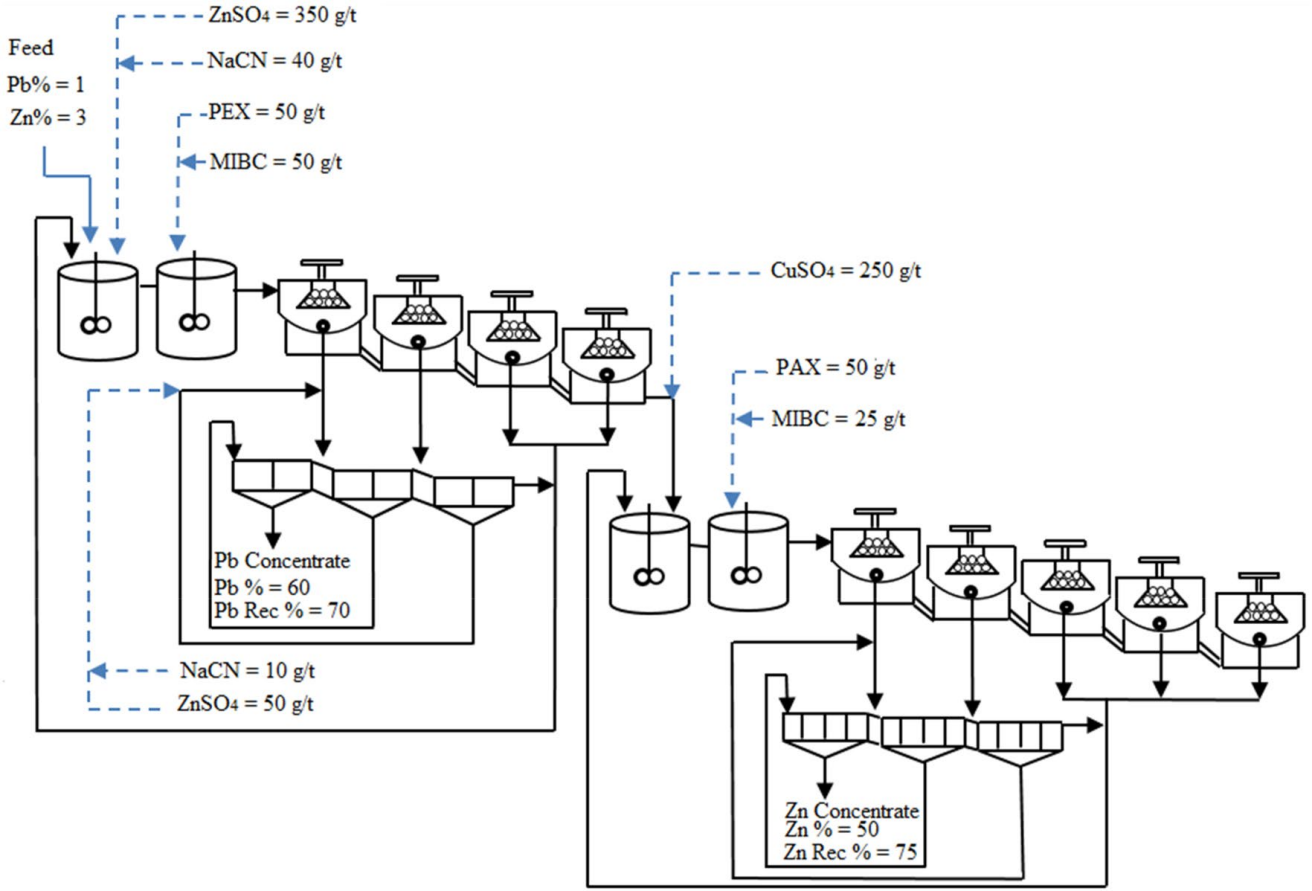
The schematic process flow diagram of actual flotation plant.

As a processing strategy, the valued minerals are separated from the gangue minerals using the comminution process (to achieve proper liberation) and then differential flotation of galena and sphalerite. In Gushfil flotation plant, galena is firstly floated in the lead circuit while sphalerite and pyrite are depressed by NaCN and ZnSO_4_. In the zinc flotation circuit, sphalerite is activated using copper sulphate and floated with specified collectors. The schematic diagram of the actual Gushfil flotation plant is shown in Fig. 1.

In both lead and zinc circuits, pyrite is the main sulphide gangue mineral. It is critical to decrease the presence of iron (Fe) and Zn in the lead concentrate during the flotation of galena. Moreover, reducing the amount of iron in the zinc concentrate is also important for making the process more economical. In addition, increasing the recovery of lead and zinc flotation will decrease the adverse impact of produced tailings on the environment.

In practice, this is not always easy due to the different behaviour of complex ores from pure single minerals. Selective flotation of sulphide minerals from complex ore is dependent on the several parameters such as: type and dosage of collectors, galvanic interactions and activation by dissolved ions^5–9^. Therefore, depressants have been used to improve the selectivity of desired minerals by acting on a mineral surface or by reacting with other reagents such as collectors and activators^10^. Chitosan has been used as a selective depressant in the differential Pb and Zn sulphide flotation on synthesized sphalerite and galena mixtures^11^. It has been shown that, after EDTA addition, sphalerite recovery has been decreased while galena has been floated at pH 4. It was shown that sphalerite could be activated by cuprous cyanide complexes in the lead flotation circuit and it leads to the copper adsorption on the sphalerite surface. It means that higher cyanide is needed to leach out copper from the sphalerite surface for the efficient depression of sphalerite^12^. It was also indicated that sphalerite can be separated from the mixture of sphalerite—galena using dextrin as a depressant for galena at pH 12^13^.

On the other hand, the pH of the pulp determines the surface charge of the minerals and adsorption of reagents onto the minerals that led to the selective flotation of minerals from the ore^14^. Hence, the pH can be adjusted to act as a simple and economical depressant to achieve higher selectivity. Also, this method has less environmental problems and is regarded as the first step in the optimization of the process^5^. For example, pyrite depression with lime at high pH values is one of the well-known operational conditions in the sulphide minerals flotation plants^15–17^.

In addition, to achieve better selectivity and recovery, reagent dosage plays a crucial role in the flotation process. Therefore reagents specially collectors and the pH should be accurately balanced to obtain optimum grade and recovery^16^. Xanthate collectors are one of the most broadly used collectors for sulphide minerals flotation, especially for easy- to- treat ores where selectivity against iron sulphides and penalty elements is not a critical problem^5,17^. Increasing the carbon chain length of xanthates increases the recovery power while lowering the selectivity^18^. On the other hand, the stability of these collectors at low pH decreases and hence they are not suitable for flotation in acidic conditions^17^. Flotation behaviour of galena and mechanism of adsorption of collector on the surface of galena after cyanide treatment has been investigated by the researchers. It was found that there is electrostatic adsorption in the interaction between butyl xanthate and the surface of galena after cyanide treatment in the pH range 4.2–8.4^19^. Therefore, to obtain desired metallurgical results, using other collector types or a combination of two or more different collectors is necessary. Phosphine-based collectors and dithiophosphates are one of the alternatives that are used in the flotation of complex minerals in combination with xanthates or used individually^17^. Aero 3418 A and Aero 3477 are suitable options for increasing the selectivity in the lead sulphide and zinc sulphide flotation circuits, respectively. Aero 3418 A has a good selectivity against iron sulphide and Aero 3477 is strong and selective for the activated zinc minerals and exhibits selectivity against the iron sulphides^17^. Therefore, the aim of this study is to find the optimum pH and collector type and dosage for the galena and sphalerite flotation from a complex lead–zinc ore.

## Materials and methods

### Materials

The lead–zinc ore was supplied by Gushfil mine, Iran. Ore samples were crushed to produce − 2 mm particles using jaw and roll crushers. Then, the samples were ground in a mild steel rod mill for a certain time to achieve particle size 80% − 75 µm. All chemicals were used at industrial grade without any further purifications. X-ray powder diffraction (GIXRD, ASENWARE AW-XDM 300) and X-ray fluorescence (XRF, Philips PW1480) were conducted on the flotation feed, lead and zinc concentrates and tailing to identify the mineral content and elemental composition. The minerals content was semi-quantitatively calculated by Rietveld-like (RIR) method. In this method, XRD and XRF analyses results were combined for determination of minerals abundance (weight percentage) in the samples^20^.

### The pulp pH and redox potential measurement

The solution pH was measured using a Metrohm 827 pH meter. The redox potential was determined with a reference to a saturated Ag/AgCl electrode using a Pt electrode attached to a Metrohm 827 pH meter^21^. The precision of the measurements was tested using a Crison standard redox solution of 250 mV at 25 °C^21^.

### Flotation experiment

The flotation tests were performed in self-aerated Denver flotation machine with a 2.3 L cell and agitation speed was adjusted to 900 rpm. The pH of pulp (5–11.5) and collector type in the flotation cell were changed according to the experimental requirements. In the lead flotation, certain amounts of depressants (zinc sulphate 400 g/ton and sodium cyanide 50 g/ton with 5 min conditioning), collector (PEX 40 g/ton or Aero3418 10 g/ton with 2 min conditioning) and frother (MIBC 20 g/ton with 1 min conditioning) were added respectively after adjusting pH to desired values (5–11.5). The pH of the pulp was monitored after adding each reagent.

**Figure 2:**
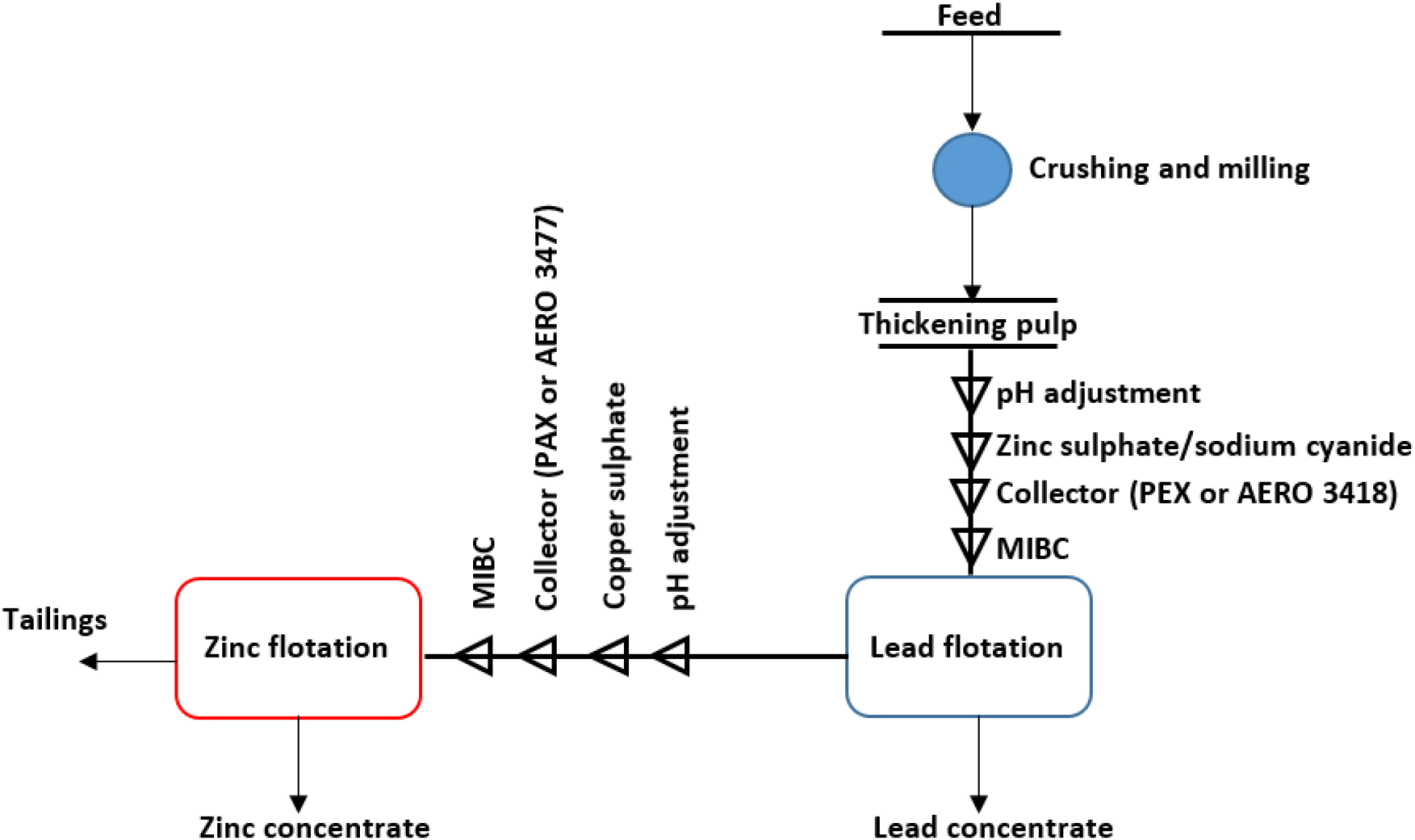
The simple flowsheet of flotation tests.

The zinc flotation tests were conducted after determination of optimum conditions for the lead floatation. In the zinc flotation experiments, after pH adjustment, conditioning was performed by adding copper sulphate as sphalerite activator (250 g/ton for 2 min), PAX (50 g/ton) or Aero 3477 (20 g/ton) as sphalerite collector for 2 min and frother (MIBC 20 g/ton for 1 min). After conditioning, the flotation was started with the injection of air in the flotation cell, while the self-aerated regulator was kept entirely open in all the flotation tests. The flotation was performed for 3 min for both lead and zinc concentrates as shown in Fig. 2, then, the flotation products were weighted and analysed chemically. Using well-known equation [Eq. (1)], recoveries (or distributions) were calculated^22^.

1 $$R = \frac{Cc}{{Ff}} \times 100$$

where C is the weight of lead or zinc concentrate and F is the weight of feed that here is 1 kg. The symbols of c and f are the grades of lead, zinc or iron in the concentrates and feed, respectively.

### Kinetics study

The kinetics experiments were conducted for both lead and zinc circuits using PEX and PAX collectors, respectively. The experiments were carried out at pH values of 6 and 8. The classical first-order flotation model as a standard model was used to find kinetics parameters of lead and zinc flotation circuits^23,24^.

2 $$R = R_{\infty } (1 - \exp ( - kt))$$

where *k* is the average flotation rate constant, *R*_∞_ denotes the ultimate recovery, R is the recovery of desired minerals and t is the flotation time.

## Results and discussion

### Mineralogical analysis

The elemental composition of the flotation feed, the lead and zinc concentrate, and tailing are provided in Table 1. As it can be seen, the amounts of lead and zinc are 0.93 wt% and 2.8 wt% in the ore. It is important to investigate the mineral content of the ore to identify which minerals contain these elements in order to select suitable collector type and operating pH. The mineral content of the flotation feed, lead and zinc concentrate, and tailing are listed in Table 2. TXRD and mineralogical studies showed that pyrite and sphalerite are the major sulphide minerals and galena is the minor sulphide mineral. Also, dolomite, barite and quartz are the major gangue minerals The oxidation rate of lead and zinc was 11 and 5 percent respectively.

**Table 1:** Elemental composition of flotation feed, lead and zinc concentrates, and tailing.

Element	Feed (wt%)	Lead concentrate (wt%)	Zinc concentrate (wt%)	Tailing (wt%)
SiO_2_	48.73	41.50	32.68	48.85
CaO	7.82	5.54	4.91	7.45
Fe_2_O_3_	4.41	4.93	4.79	4.56
K_2_O	2.28	2.29	1.57	2.13
MgO	5.55	4.04	3.51	5.33
Al_2_O_3_	7.02	4.12	3.98	7.91
Na_2_O	4.89	3.04	2.94	6.93
MnO	0.28	0.21	0.18	0.27
Ba	0.41	0.30	0.25	0.36
S	2.50	4.26	13.73	1.06
Pb	0.93	11.22	–	–
Zn	2.80	3.00	25.37	–

**Table 2:** Mineral content of flotation feed, lead and zinc concentrate, and tailing.

Mineral	Feed (wt%)	Lead concentrate (wt%)	Zinc concentrate (wt%)	Tailing (wt%)
Quartz (SiO_2_)	37	31	26	37
Mica-illite (KAl_2_Si_3_AlO_10_(OH)_2_	22	19	13	23
Dolomite (CaMg(CO_3_)_2_	21	17	11	22
Albite (NaAlSi_3_O_8_)	2	2	1	4
Chlorite (Mg,Fe)_6_(Si,Al)_4_O_10_(OH)_8_	8	7	6	8
Sphalerite (ZnS)	5	5	39	–
Pyrite (FeS2)	3	2	2	2
Galena (PbS)	1	15	–	–
Other	1	1	2	4

**Figure 3:**
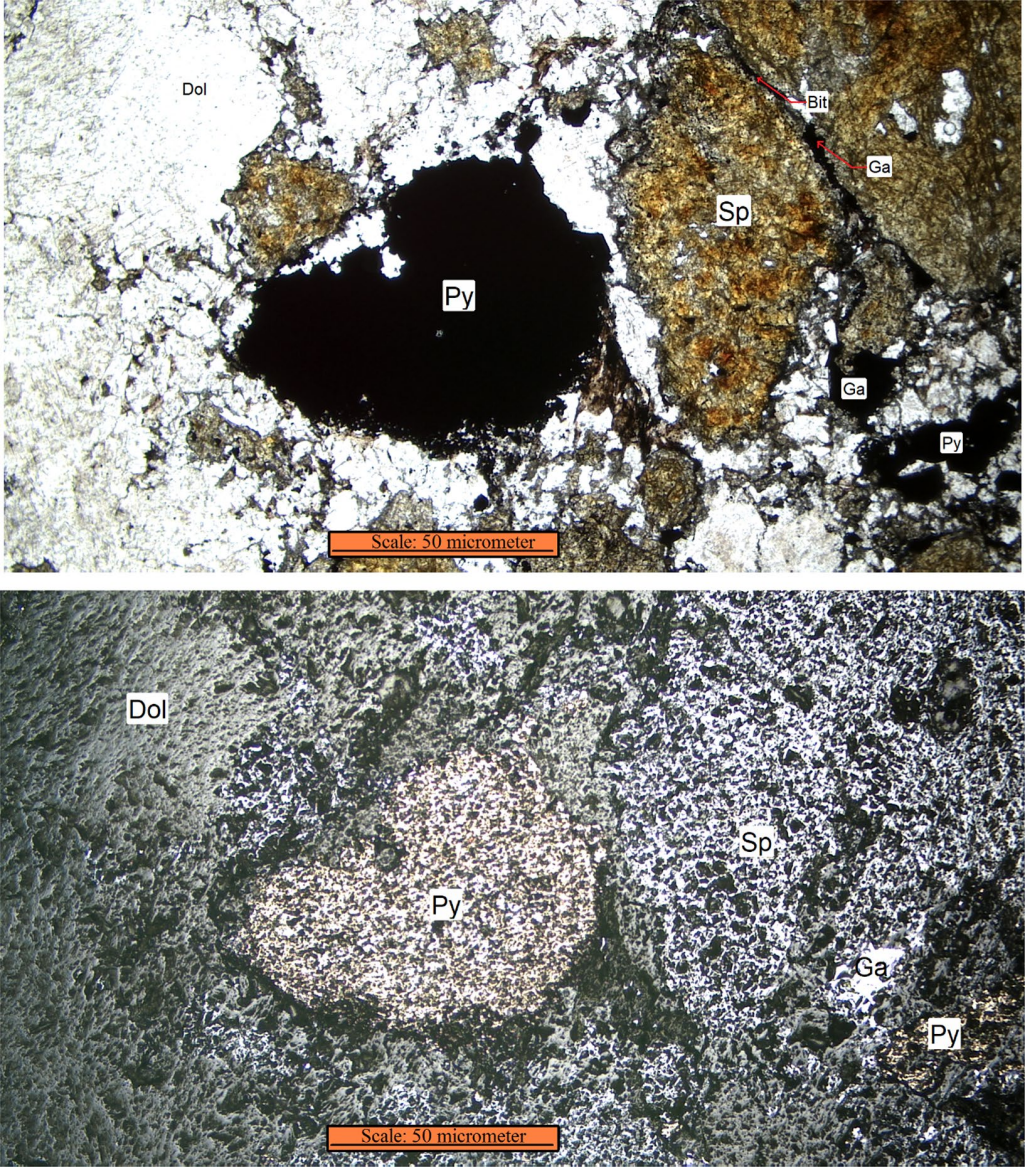
Microscopic image of a thin section from a fragment of Gushfil ore, transmitted image (up), reflected image (down). The showed minerals are sphalerite (Sp), galena (Ga), pyrite (Py), dolomite (Do), bitumen (Bit).

Transmitted (up) and reflected (down) microscopic images of the run of mine ore cross-section are shown in Fig. 3. Crystals of sphalerite, galena, and pyrite as sulphide minerals and dolomite as the main gangue mineral can be seen in Fig. 3. Some of the cracks are filled with galena and a tiny amount of bitumen minerals.

### Lead flotation stage

#### Effect of pH on the grade and recovery of lead

**Figure 4:**
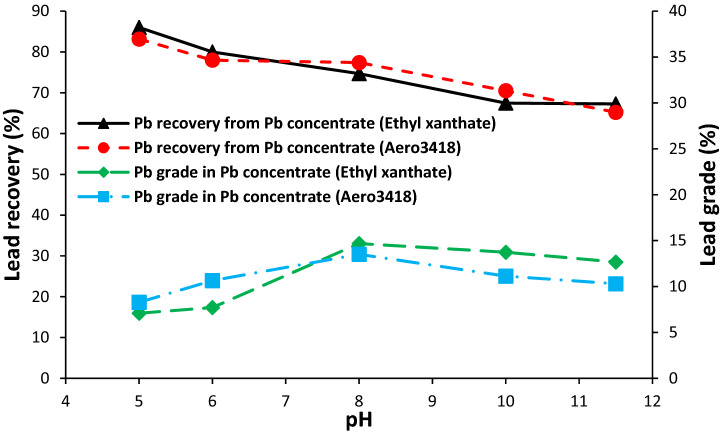
Effect of pH and collector type on the Pb recovery and grade.

**Figure 5:**
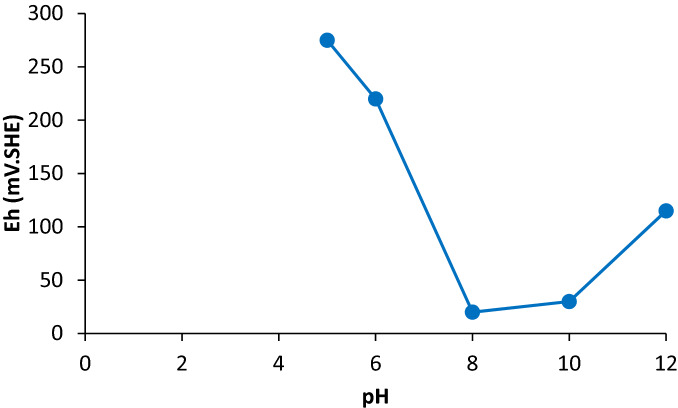
Eh (mV.SHE) vs. pH after pH adjustment and before adding chemicals in the pulp for the lead flotation.

PEX and Aero 3418 were used as collector. The PEX is a common collector and is used in the Gushfil plant for the galena flotation. The Aero 3418 was selected because not only it has lower affinity toward depressed zinc sulphide, but also it has lower selectivity for the iron sulphide. Therefore, the effect of these collectors on the lead recovery was investigated at different pH values. The results of the lead flotation using PEX and Aero 3418 A at various amounts of pH was shown in Fig. 4. As can be seen, the lead grade approached the highest value at pH 8 and decreased at higher pH values for both collectors, while, the lead recovery decreased with increasing the pH. As Fig. 5 shows a substantial increase was observed in the pulp redox potential when the pH decreases from 8 to 5 which can facilitate lead recovery^25^. It has been reported that the flotation of the pure galena takes place at pH values in the range of 2–10^26^. The formation of plumbite instead of lead xanthate has been reported as a reason for the reduction of lead recovery at pH values higher than 11^18^. Also, It has been shown that, Pb(OH)^+^ adsorbed on the galena or sphalerite surface can reduce the collector ability for the galena flotation in the pH values between 7 and 9.5, but had a significant activating effect on the sphalerite floatability in the pH range 7.5–10.5^27^. The lead flotation in the industrial scale is usually carried out in the pH range 7 to 10.5 based on the ore mineralogy and petrogenesis^16^. But, as the lead grade is an important consideration, other impurities such as zinc and iron in the lead concentrate should be reduced.

#### Zinc and iron distribution and grade in the lead concentrate

**Figure 6:**
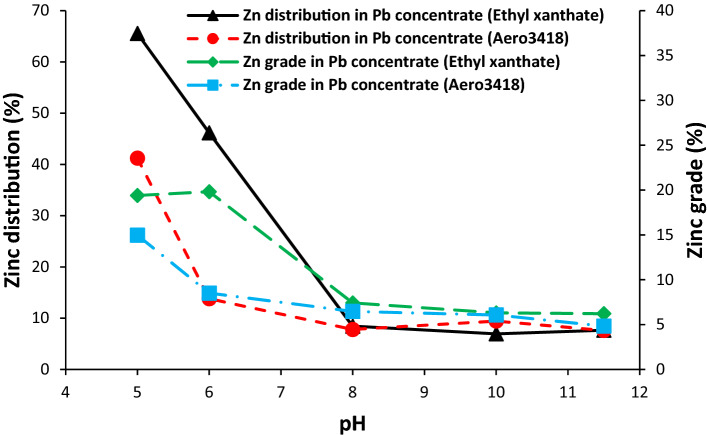
Effect of pH and collector type on the distribution and grade of zinc in the lead concentrate.

The influence of pH and collector type on the distribution of zinc in the lead concentrate is shown in Fig. 6. For both collectors, it was observed that the zinc distribution in the lead concentrate and its grade has a downward trend, when the pH was increased from 5 to 8. In spite of higher lead recovery at pH 5 (Fig. 4), the distribution of zinc in the lead concentrate is also higher which causes unacceptable losses of zinc in zinc concentrate. The pH value of 8 is the optimum pH for the lead flotation because the lead grade is in its maximum value and the zinc distribution in the lead concentrate is low.

**Figure 7:**
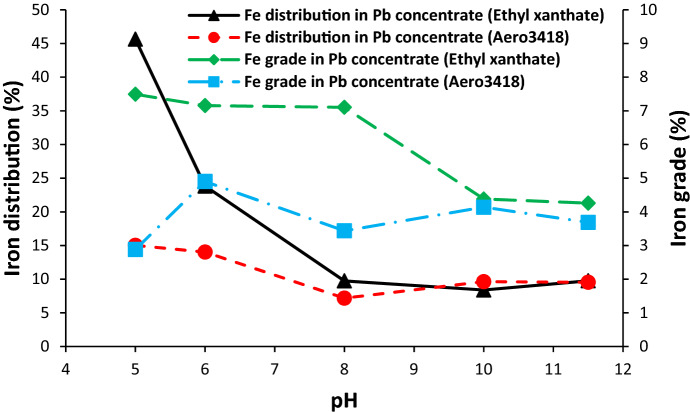
Effect of pH and collector type on the iron distribution and grade in the lead concentrate.

The effect of pulp pH on the distribution and grade of iron in the lead concentrate is shown in Fig. 7. It is clearly seen that the distribution and grade of iron in the lead concentrate are lower than 10% for both collectors at pH 8. It means that the iron distribution and grade is in the acceptable range for the flotation of lead. Also, Aero 3418 collector showed suitable behaviour against iron and zinc at lower pH values.

As can be seen from Figs. 6 and 7, the distribution of zinc and iron in the lead concentrate increases with decreasing pH. It should be attributed to the decreasing of cyanide ion (CN^−^) in the pulp at lower pH value. The cyanide ion has a depressing effect on the zinc and iron sulphide^15,18^. In the pH range of 6 to 8.4, cyanide is volatilized and more than 90% of that occurs as hydrogen cyanide in the pulp that leads to cyanide losses from the pulp^28^.

### Zinc flotation stage

#### Influence of pH and collector type on the recovery and grade of zinc

**Figure 8:**
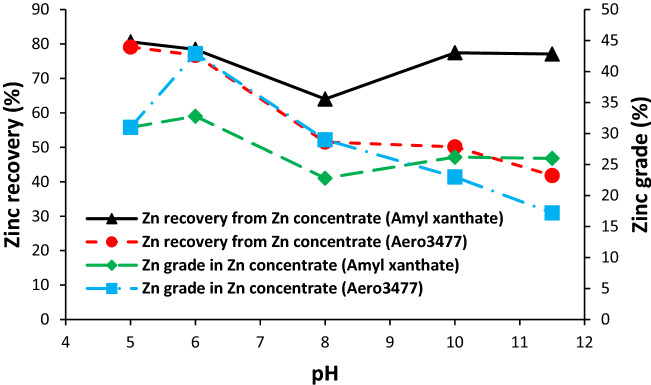
Effect of pH and collector type on zinc recovery and grade in the zinc concentrate.

After lead flotation at optimum pH 8, the flotation of zinc at a different pH (5–11.5), with two collector types, was performed to study the recovery and grade of zinc in the zinc concentrate. Figure 8 shows the grade and recovery of zinc in the zinc concentrate using PAX or Aero 3477 collectors at pH 5–11.5. It can be observed that the maximum grade and recovery for zinc were obtained at pH 6 for both collectors. The grade and recovery of zinc were decreased from 43 to 17% and from 77 to 42% with increasing pH from 6 to 11.5 in the presence of Aero 3477 in the pulp. Hence the use of Aero 3477 at lower pH values is recommended. Zinc grade and recovery reduced in the pH range of 6–8 from 33 to 23% and from 78.5% to 64%, respectively, when PAX was used as collector, however, there is an increase in grade and recovery from 23% to 26.2% and from 64 to 77% at pH 8 to 11.5. Fuerstenau et al.^18^ reported that the maximum recovery of sphalerite in the presence of PAX takes place at pH 3.5 and it decreases with increasing pH. The increase in the zinc recovery at pH values in the range of 8–11.5 in this study can be due to the addition of copper sulphate as sphalerite activator in the pulp which has been reported by Hu et al.^25^. They reported that in absence of copper sulphate, sphalerite recovery decreases gradually from pH 3 to 8 with butyl xanthate as collector but In the presence of copper sulphate, recovery of sphalerite can be higher than 90% at pH < 12^25^ which is in agreement with our results.

#### Iron distribution and grade in the zinc concentrate

**Figure 9:**
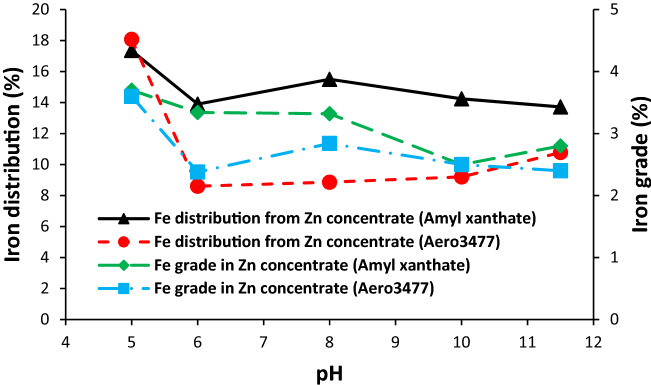
Effect of pH and collector type on the iron distribution and grade in the zinc concentrate.

Iron distribution and grade in the zinc concentrate are presented in Fig. 9. The lowest iron distribution was observed for both collectors at pH 6. On the other hand, at this pH, the grade and distribution of zinc were maximum (Fig. 9).

### Flotation kinetics rates of lead, zinc, and iron

**Figure 10:**
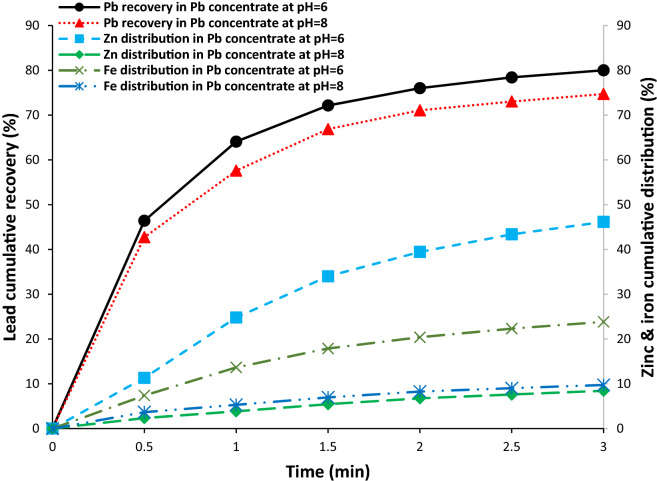
Flotation kinetic of lead, zinc, and iron at pH of 6 and 8 in the lead flotation stage.

The pH values of 6 and 8 were selected as optimum pH for the lead and zinc flotation respectively. Lead, zinc, and iron flotation kinetic rates in the lead flotation stage at pH values of 6 and 8 were shown in Fig. 10. As it can be seen, there is a sharp increase in the lead recovery in the first minute and then the lead recovery gradually increases from about 60% to 80%.

The average flotation rate constant (k) and the ultimate recovery (R_∞_) for the lead, zinc, and iron are shown in Table 3. Changes in pH do not have a considerable effect on the average flotation rate constant of lead, but, the ultimate recovery is 5% higher at pH 6. On the other hand, zinc and iron distribution are 46.28% and 16.47% at pH 6. It means, it is not logical to use this pH for the lead flotation. Moreover, to increase the recovery in the lead circuit flotation at pH 6, more zinc sulphate and sodium cyanide should be used to depress the zinc and iron sulphide.

**Table 3:** Flotation kinetics rate parameters for lead, zinc, and iron in the lead flotation stage.

	pH 6		pH 8	
	k	R_∞_	k	R_∞_
Pb in the Pb concentrate	1.69	79.36	1.60	74.31
Fe in the Pb concentrate	0.68	27.48	0.69	11.00
Zn in the Pb concentrate	0.55	58.25	0.41	11.97

**Figure 11:**
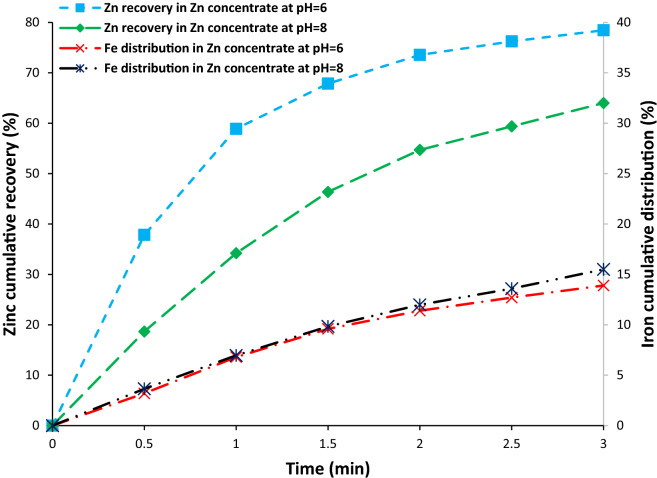
Flotation kinetic of zinc, and iron at pH of 6 and 8 in the zinc flotation stage.

Figure 11 shows flotation kinetic rates of zinc and iron in the zinc flotation stage. The average flotation rate constant (k) and the ultimate recovery (R_∞_) were tabulated in Table 3. According to Table 4, the average flotation rate constant of zinc at pH 6 is more than 2 times its value at pH 8. The ultimate recovery was achieved to be 79.56% at pH 6 while it was 77.67% at pH 8. This indicates that for cell design purposes, pH should be considered. As shown here, more recovery could be achievable with lower cell volumes due to the higher kinetics rate at pH 6. Kinetics parameters of iron are relatively similar at pH 6 and 8.

**Table 4:** Flotation kinetics rate parameters for zinc, and iron in the zinc flotation stage.

	pH 6		pH 8	
	k	R_∞_	k	R_∞_
Zn in the Zn concentrate	1.31	79.56	0.59	77.67
Fe in the Zn concentrate	0.44	19.15	0.39	21.9

## Conclusion

Using optimized pH in the lead flotation stage can remarkably improve the grade and recovery of lead, and prevents zinc losses in the lead concentrate and decreases iron content in it. The distribution of zinc and iron increases at acidic pH (below 7) in the lead concentrate. A huge reduction of iron and zinc grade and distribution at pH 8 in the lead concentrate was observed. So, this pH was considered as optimum pH for the lead flotation due to higher grade and proper recovery of lead. Aero 3418 A collector showed more selective behaviour for the zinc and iron in comparison with potassium ethyl xanthate in the lead flotation stage. In the zinc flotation stage, the grade and recovery of zinc were more acceptable at pH 6 compared with other pH values. Aero 3477 had acceptable performance at low pH values (5–7). It was shown that the pH of pulp could affect the flotation kinetics of all floatable sulphides and should be considered in the designing purposes. The results of the current study not only can improve the economical and metallurgical aspects of the process but also would solve the operational problems created in the process when working with basic pH in the zinc circuit like preparing limewater and precipitation in the tubes.
