# Author Correction: Critical importance of pH and collector type on the flotation of sphalerite and galena from a low-grade lead–zinc ore

**DOI:** 10.1038/s41598-021-91063-z

**Published:** 2021-05-26

**Authors:** Abdolrahim Foroutan, Majid Abbas Zadeh Haji Abadi, Yaser Kianinia, Mahdi Ghadiri

**Affiliations:** 1 Department of Research and Development, Bama Mining Company, Isfahan, 81747‑54754 Iran; 2 Institute of Research and Development, Duy Tan University, Da Nang, 550000 Viet Nam; 3 The Faculty of Environment and Chemical Engineering, Duy Tan University, Da Nang, 550000 Viet Nam

Correction to: *Scientific Reports* https://doi.org/10.1038/s41598-021-82759-3, published online 04 February 2021

This Article contains errors, where the tailing stream is missing in Figure 1. The correct Figure 1 appears below.

**Figure 1:**
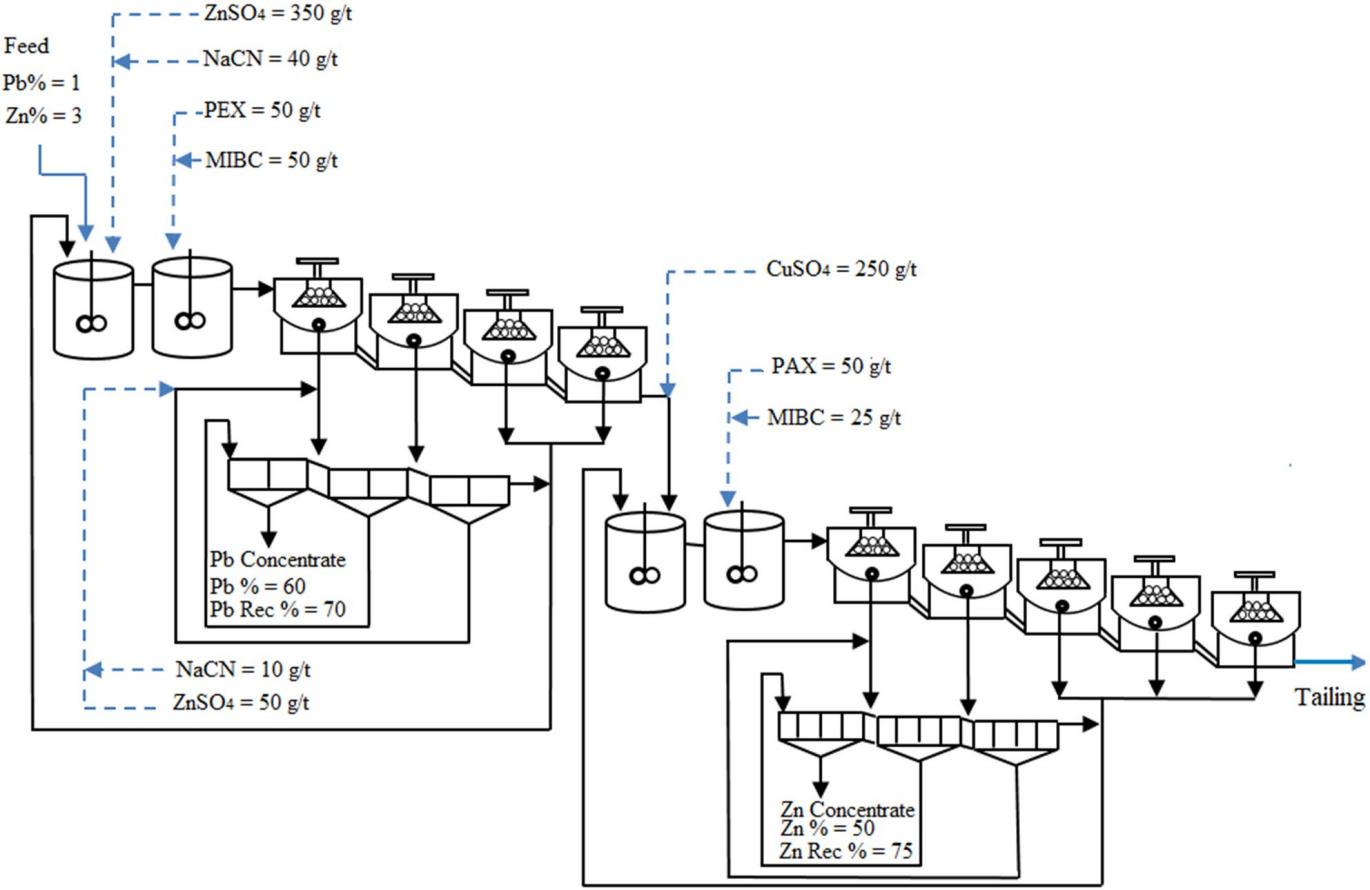
A correct version of the original Figure 1.

In addition, in Table 1, the amount of Na_2_O in Feed, Lead concentrate, Zinc concentrate and Tailing is incorrect. The correct values appear in Table 1 below.

**Table 1:** A correct version of the original Table 1.

Element	Feed (wt.%)	Lead concentrate (wt.%)	Zinc concentrate (wt.%)	Tailing (wt.%)
SiO_2_	48.73	41.50	32.68	48.85
CaO	7.82	5.54	4.91	7.45
Fe_2_O_3_	4.41	4.93	4.79	4.56
K_2_O	2.28	2.29	1.57	2.13
MgO	5.55	4.04	3.51	5.33
Al_2_O_3_	7.02	4.12	3.98	7.91
Na_2_O	0.49	0.31	0.30	0.69
MnO	0.28	0.21	0.18	0.27
Ba	0.41	0.30	0.25	0.36
S	2.50	4.26	13.73	1.06
Pb	0.93	11.22	–	–
Zn	2.80	3.00	25.37	–

In the Results and discussion section,

“TXRD and mineralogical studies showed that pyrite and sphalerite are the major sulphide minerals and galena is the minor sulphide mineral. Also, dolomite, barite and quartz are the major gangue minerals The oxidation rate of lead and zinc was 11 and 5 percent respectively.”

should read:

“XRD and mineralogical studies showed that pyrite and sphalerite are the major sulphide minerals and galena is the minor sulphide mineral. Also, dolomite, barite and quartz are the major gangue minerals The oxidation rate of lead and zinc was 11 and 5 percent respectively.”

And,

“Transmitted (up) and reflected (down) microscopic images of the run of mine ore cross-section are shown in Fig. 3. Crystals of sphalerite, galena, and pyrite as sulphide minerals and dolomite as the main gangue mineral can be seen in Fig. 3.”

should read:

“Transmitted (up) and reflected (down) microscopic images of the run of mine ore thin section are shown in Fig. 3. Crystals of sphalerite, galena, and pyrite as sulphide minerals and dolomite as the main gangue mineral can be seen in Fig. 3.”

